# Development of social skills in children: neural and behavioral evidence for the elaboration of cognitive models

**DOI:** 10.3389/fnins.2015.00333

**Published:** 2015-09-29

**Authors:** Patricia Soto-Icaza, Francisco Aboitiz, Pablo Billeke

**Affiliations:** ^1^Laboratorio de Neurociencias Cognitivas, Departamento de Psiquiatría, Facultad de Medicina, Pontificia Universidad Católica de ChileSantiago, Chile; ^2^Centro Interdisciplinario de Neurociencia, Pontificia Universidad Católica de ChileSantiago, Chile; ^3^División de Neurociencia, Centro de Investigación en Complejidad Social, Facultad de Gobierno, Universidad del DesarrolloSantiago, Chile

**Keywords:** social skills, social cognition, development, childhood, Autism Spectrum Disorders, EEG, fMRI, social brain

## Abstract

Social skills refer to a wide group of abilities that allow us to interact and communicate with others. Children learn how to solve social situations by predicting and understanding other's behaviors. The way in which humans learn to interact successfully with others encompasses a complex interaction between neural, behavioral, and environmental elements. These have a role in the accomplishment of positive developmental outcomes, including peer acceptance, academic achievement, and mental health. All these social abilities depend on widespread brain networks that are recently being studied by neuroscience. In this paper, we will first review the studies on this topic, aiming to clarify the behavioral and neural mechanisms related to the acquisition of social skills during infancy and their appearance in time. Second, we will briefly describe how developmental diseases like Autism Spectrum Disorders (ASD) can inform about the neurobiological mechanisms of social skills. We finally sketch a general framework for the elaboration of cognitive models in order to facilitate the comprehension of human social development.

## Introduction

Social cognition involves all the abilities that enable us to understand social agents and to interact with them. In this process, it is crucial to be able to predict the behavior of others, by detecting, analyzing, and interpreting their intentions. In this paper, we adopt a developmental perspective to clarify how social understanding evolves (Rao et al., 2008; Alaerts et al., 2011). For instance, during social development it is possible to observe social behavior precursors, which are necessary abilities for developing the capacity to deal with more complex social information (i.e., to deal with a group of people). Social skills, such as the detection of biology motion and sensitivity to eye-like stimulus, can be understood as precursors, not only because they appear first in human life but also because they are required for the acquisition of further social abilities, like face recognition or joint attention (Charman et al., 2001; Happé and Frith, 2014). Thus, these social precursors form a temporal sequence of events that may be needed to give rise to appropriate social behavior. Indeed, prior work has shown that the development of social skills encompasses a complex and delicate interaction between several elements, such as smiling, eye contact, imitation, joint attention, language, and the observer's own motor system among others. These elements play a role in the accomplishment of positive developmental outcomes, including peer acceptance, academic achievement, and mental health (Rao et al., 2008). Although this temporal sequence encompasses changes that can be observed at both neural and behavioral levels, the literature about social development has drawn up different concepts over the years. These concepts have been elaborated to construct cognitive models of social functioning that can explain the connection between behavior and brain mechanisms (Johnson, 2011). Even though in the literature the social cognition concept is commonly used as a good fit for this connection, an overview of social phenomena includes several concepts that interact and overlap each other, such as the social brain, social cognition, social behavior, and social functioning (Baars and Gage, 2012; Billeke et al., 2013a). We summarize some of the key concepts in Table 1.

**Table 1:** **Social concepts**.

**Concept**	**Definition**	**Level involved**		
		**Neural**	**Cognitive**	**Behavioral**
Social brain	Brain network whose function is associated with social processing. It could be described as structures operating in a network that could enable an accurately social performance			
Social cognition	All kind of cognitive processes that can allow us to interact with others and to understand other people's intentions, feelings, emotions, and behaviors			
Social behavior	The ability to interact with others			
Social skills	A wide group of abilities that emerges from the appropriate execution of social cognition processing. This adequate performance allows us to interact and communicate with others, by predicting and understanding other people's intentions, feelings, emotions, and behaviors			
Social functioning	Social behavior when it is integrated over time and context			
Social precursors	A group of very early onset abilities readily observable in newborns or early infancy such as eye-like sensitivity, biological motion preference, and imitation			

*Colors represent the different levels of description that involved each concept. Green indicates neural level, gray cognitive level and orange behavioral level*.

Following the main contributions in this area, we will describe the most important evidence for the development of social skills at three levels, namely neuronal, cognitive, and behaviorally. While the neuronal and behavioral levels are an aspects that can be directly observed and measured, the cognitive one considers different models about how neuronal mechanisms lead to behavior (i.e., the way in which the brain is associated with behavior). Thus, we will organize this review in three sections. We will first make a timeline of the behavioral events that may be related to social development. Then we will draw a chronology of the appearance of neural and cerebral events that have been linked to these social behaviors. Second, we briefly review how conditions that involve primary social impairments, like Autism Spectrum Disorders (ASD), can inform about both the trajectory of social development and the neurobiological mechanisms related to the social behavior. Finally, we will sketch a general framework for the elaboration of cognitive models in order to facilitate the comprehension of human social development.

## Development of social behavior

Consistent evidence has reported that the abilities associated with visual processing are crucial for the development of social skills (Emery, 2000; Happé and Frith, 2014). These studies have shown that capacities such as eye-like sensitivity, biological motion preference, imitation, face recognition, and gaze following are present from the very beginning of human life (Courchesne et al., 1981; Emery, 2000; Webb and Nelson, 2001; Itier, 2004; Dalton et al., 2005; Csibra et al., 2008; Hoehl et al., 2008; Baars and Gage, 2010; Billeke and Aboitiz, 2013; Happé and Frith, 2014; Peña et al., 2014; Von dem Hagen et al., 2014). These abilities could be understood as the first signs of social capacities, which later should deal with more complex stimulus and social interactions (i.e., to discriminate among familiar and unfamiliar faces, to initiate joint attention, etc.).

### Social agent detection: early eye-like sensitivity, imitation, and biological motion preference

From a very early age, human and non-human primates show a set of visual behaviors that seem to influence social development. Specifically, preference for focusing on eyes (eye-like sensitivity) and eye-like stimuli have been widely described in the literature as having a crucial function for social development. Studies in non-human primates have shown that head and eye orientation can provide crucial signals to the understanding of the social world (Emery, 2000). Coincidently with this, a recent study in human infants showed that typically developing (TD) children from 2 to 6 months of age look more into the eyes than at mouth and body (Jones and Klin, 2013). This research also reveals that eye fixation increases from 2 to 24 months of age, showing that human social engagement may be related to the this visual capacity already present in such early age.

The eye-like sensitivity also provides the possibility to learn from others, which is an essential task of the developing social brain (Gariépy et al., 2014; Happé and Frith, 2014). A landmark study revealed that 12 days old infants have a mimicry behavior (Meltzoff and Moore, 1977). This imitation behavior occurred for the four gestures that were assessed, namely lip protrusion, mouth opening, tongue protrusion and sequential finger movement performances by an unfamiliar experimenter. The fact that infants imitate not one, but four different gestures, support the interpretation that basic imitation might be an innate ability. In accordance to this, ethological studies have revealed that early imitation is also present in non-human primates (Ferrari et al., 2006; Paukner et al., 2011). A study with infant macaques showed that the imitative responses are already present since the first day of life, when infant macaques are able to imitate lip smacking, elicited by a model's mouth opening, and tongue protrusion, showing a phylogenetic aspect of human behavior (García et al., 2014). Interestingly, the neonatal imitation of the lip smacking may sub-serve for infants' affiliative responses to the social world, because this behavior is a core gesture in face-to-face interaction in macaques (García et al., 2014).

The detection of social agents, however, depends not only on eye-like sensitivity and gaze following, but also relies on another crucial visual ability, namely, the biological motion discrimination. Biological motion refers to the remarkable capacity to discriminate and recognize biological motion patterns as a set of moving dots on the main joints of an invisible walker. Actually, several findings have revealed that despite the perceptual ambiguity that this experimental stimulus may involve, humans readily extract the invariant structure from biological motion (Pavlova and Sokolov, 2000). The mechanisms through which humans can interpret the complex sequences of action of other humans has been a topic of interest to researchers for decades (Johnson, 2006). Since the landmark study by Johansson (1973), several studies have shown that human beings are able to identify body motion directions as well as to discriminate different kinds of limb motion patterns (Johansson, 1973; Bertenthal et al., 1984; Pavlova and Sokolov, 2000; Simion et al., 2008). Similarly to eye-like sensitivity, biological motion preference might be also an early ability, because newborn human beings are able to discriminate biological motion from non-biological motion (Simion et al., 2008). Newborns aged 1–3 days were able to discriminate between a biological motion animation (i.e., moving array of point-lights attached to the joints of an individual during a walk) and a non-biological motion animation sequence (i.e., the random motion), which is reflected in the longer fixation time to these stimuli (Simion et al., 2008). Indeed, their findings also revealed that this biological motion preference is also orientation-dependent, because newborns looked longer at upright arrays than at inverted biological motion displays. Interestingly, other study found that infants of 3 and 5 months present these preferences only for moving displays and not with the static arrays (Bertenthal et al., 1984). Furthermore, this effect was not found to interact with the age of the infants or the upright or inverted form. Recently, it has been shown that infants, as young as 12 months old, can follow the direction of point-light moving array with the gaze (Furuhata and Shirai, 2015). This reveals a continuous development and specialization of the ability to discriminate biological motion, probably influenced by experience of environmental exposition.

### Toward a shared world: gaze following ability and face recognition processing

Following the idea that social development is a set of concatenated elements, it should be noted that the eye-like sensitivity seems to precede the posterior ability of gaze following. A recent study in full-term and preterm infants showed that visual experience has a significant influence on the development of early gaze following (Peña et al., 2014). By using eye tracking, they found that gaze following in preterm infants was similar to that of full-term infants with the same chronological age, despite their difference in postmenstrual age. This fact highlights the importance of the environmental stimulation for the development of this ability. The undeniable participation of the environment is also present in non-human primates. Shepherd et al. (2006) demonstrated that in low-status male rhesus macaques, the gaze following was a reflexive process, while in high-status macaques the gaze was a voluntary mechanism. By using a simple visual orienting task paradigm, they showed that in low-status macaques the reaction time for saccades made to a peripheral target after viewing an image of a familiar monkey in that direction was faster than in high-status subjects. Even more, high-status macaques showed a complete lack of inhibition of return of the saccade. According to the authors, these findings reveal that faster gaze following and later inhibition of return in low-status monkeys involves a reflexive attention, whereas in high-status monkeys lower gaze following and absence of inhibition of return implies a voluntary component of attention.

As we discussed above, it is important to consider that eye-like sensitivity and gaze following are related to another crucial ability, namely face recognition. Several studies have shown that the ability to recognize faces specializes over time (Johnson, 2011; di Giorgio et al., 2012; Zieber et al., 2013; Macchi Cassia et al., 2014). Kelly et al. (2005) proved that newborn infants did not show any spontaneous preference for faces from either their own- or other-ethnic groups, however, 3-month-old infants did show a clear preference for faces from their own-ethnic group. Thus, the influence of the environmental experience during the first 3 months of postnatal life is plenty enough for inducing a visual preference for own-race faces. Turati et al. (2005) demonstrated that infants of the same age display a spontaneous visual preference for an upright image of a real face over an upside-down version of the same face. Furthermore, the distribution of looking times indicates that infants looked longer toward the eye area of the face, although only in the case of the upright face configuration (Turati et al., 2005; Jones and Klin, 2013). Thus, eyes are not strong enough cues to attract the gaze of infants of this age, because their interest is modulated by the context in which the eyes are located (Turati et al., 2005). In addition, Quinn et al. (2002) tested the perception of gender of human faces in 3- and 4-month-old infants and proved that infants were able to discriminate among female and male faces. They familiarized one group of infants with female faces and another group of infants with male faces in order to assess the ability to discriminate a member within a given category (male or female). Their results showed that the group that was familiarized with female faces exhibited a preference for a novel female face, while the group of infants that were familiarized with male faces revealed a preference for a novel male face (Quinn et al., 2002). Taken together, these findings suggest that the age of 3 months may represent a milestone for face processing, revealing that the first signs of cognitive specialization for faces are present around this age (Kelly et al., 2005; Turati et al., 2005; Johnson, 2011; di Giorgio et al., 2012; Zieber et al., 2013; Macchi Cassia et al., 2014). In this context, the relative weight of the genetic and environmental influences over this process remain unclear (Turati et al., 2005).

### Reaching the understanding of others: joint attention, social perspective-taking, and theory of mind

Even though the evidence reveals that apes can understand the expression of signs like posture, vocalization, and facial expression and are able to take action based on those signs, the capacity to understand the subjectivity of another member of their own species is an ability highly developed in humans (Baars and Gage, 2010). Considering the evidence reviewed in the previous section, it can be argued that the development in humans of the ability to detect social agents relies on the development of gaze abilities. Moreover, following the temporal sequence of social behaviors it is possible to state that, at the beginning, early preference to look at faces in mutual gaze (Farroni et al., 2002) is revealing a preference to a social situation where the visual attention of two individuals is directed at each other (Emery, 2000). Secondly, this basic level of complexity evolves to a complex level where the earlier capacity of gaze following (Farroni et al., 2002; Hoehl et al., 2008; Jones and Klin, 2013) allows children to develop the ability to identify that the glance of their partner is focusing away from them and, thus, direct their own attention toward the partner's focus of attention. This time sequence could end in the development of an even more complex ability which now includes a third element, namely joint attention. Interestingly, earlier levels of social development such as mutual gaze and gaze following, include a dyadic social relationship, but joint attention ability implies a triadic communication. Joint attention (JA) ability is defined as the capacity to share the perception of a common object with another person (Mundy et al., 2000; Charman et al., 2001; Charman, 2003; Morgan et al., 2003; Striano et al., 2006; Lachat et al., 2012; Hopkins and Taglialatela, 2013). A key component of JA is the division and the alternation of the subject's attention between the object and the partner (Bakeman and Adamson, 1984; Charman, 2003; Striano et al., 2006). The two most common behaviors of JA are pointing to designate interest in an object and alternating eye gaze to check that both the child and the partner are attending to the same event (Morgan et al., 2003). Several studies agree that JA emerges around the age of 9 months (Morgan et al., 2003; Striano et al., 2006; Kopp and Lindenberger, 2011), when children learn to use eye contact to derive information about another person's goal-directed behavior (Morgan et al., 2003). Joint attention ability can be dissociated in mainly two types of behavior: responding JA and initiating JA (Mundy et al., 2000, 2009; Mundy and Jarrold, 2010). The first one refers to the case where the child responds to gestures that a communicative partner produces (Mundy et al., 2000; Hopkins and Taglialatela, 2013). The second one refers to the case where the child spontaneously points, shows, and uses eye contact to share the experience of an object (Mundy et al., 2000). The initiation of JA behaviors seems to appear later in development than the capacity to respond to JA (Hopkins and Taglialatela, 2013).

Another social skill that contributes to the development of social knowledge is the social perspective-taking, which according to Moll and Kadipasaoglu (2013) emerges after the development of joint attention. It is important to notice that this new social ability has to deal with the problem of generating more accurate expectations or predictions about the other's behaviors (Koster-Hale and Saxe, 2013; Billeke et al., 2015). The social perspective-taking refers to some level of comprehension of other's purposes, objectives and preferences. Actually, it is possible to suggest that around 24 months of age infants appear to be able to identify other's references and perspectives. At this age, children can capture previous expressions of other's preference in order to contrast new experience with this previous experiential background. In this way, infants can identify the difference and interpret others' intentions or preferences. This background is made up by what the child and his communicational partner did, witnessed, or heard. Thus, this experiential background provides a template that allows establishing a social reference and finally a social perspective-taking. This ability has been evidenced by means of tasks that measure implicit mentalization (Southgate et al., 2007; Surian et al., 2007; Baillargeon et al., 2010), for example, the object detection task, which is a variant of the standard false belief task (Kovács et al., 2010). This experimental design generally uses an agent who is looking for an object of interest that was left in a place which both, the infant and the agent know, or that only the infant knows. For instance, an interesting study carried out by Surian et al. (2007) showed that 13-month-old infants looked longer to a non-familiarized stimulus only if it is in the agent's visual field. This finding reveals that infants take into account the agent's visual perspective to generate different expectations about the agent's future actions. Coincidently, a recent study demonstrates that at this age, infants are able to understand other people's interactions, revealing a preverbal theory of mind ability (Choi and Luo, 2015). This study assessed the responses to a false belief paradigm, showing that infants looked reliably longer to the scene where a puppet reacted in a positive manner to the agent that had previously hit another puppet. These results evidence that infants might keep some sort of record of past negative interactions and are able to associate them with the identity of the person having shown aggressiveness. Thus, the support for the infant's perspective comes from the data on false belief of the puppet who does not know that the now friendly puppet was previously rude.

The above evidence indicates that the infant's gaze can be a useful experimental tool to assess the ability to predict other people's behavior in preverbal children. In fact, Southgate et al. (2007) showed that a predictive looking paradigm allows measuring the child's expectation of where the agent will be going to look for his/her goal-object. In their study, the authors interestingly found that 25-month-old infants remained attentive to the area where the agent is expected to look for if he/she had a false belief. Therefore, the standard false believe test suggests that the ability to understand or at least to perceive the belief states of other individuals is present at ages earlier than 4 years.

However, the explicit skill to identify other people's false beliefs becomes evident only in 4-year-old children (Perner and Roessler, 2012). Premack and Woodruff (1978) presented theory of mind as a social skill that refers the ability that allows the individual to be able to assign mental states to himself/herself or to others, such as purpose, intention, knowledge, belief, thinking, doubt, guessing, pretending, feeling, etc. According to the time sequence of social behavior described here, several studies suggest that a precursor of this theory of mind mechanism could be the ability of joint attention (Baron-Cohen et al., 1985; Charman et al., 2001) and social perspective-taking. In fact, Moll and Kadipasaoglu (2013) state that social perspective-tacking emerges between the development of joint attention and theory of mind. The theory of mind ability can be considered as a stage of cognitive development that reflects the child's understanding that minds are not just copies of reality, but representations that could be true or false (Tager-Flusberg, 1999). In this regard, the evidence that comes from the false belief task is a robust indication that this is a major landmark in social development (Baron-Cohen et al., 1985; Wellman et al., 2001). The mechanism by which the switch to an explicit verbalization to other people's perspective occurs is still debated. Some authors argue that the development of cognitive abilities related to language and response inhibition are reflected in the explicit theory of mind (Baillargeon et al., 2010). By contrast, the correlation between explicit perspective taking test and classical false belief task can be used to claim that the explicit theory of mind reflect an intentional switch of perspective that it is not possible before 4 years of age (Perner and Roessler, 2012).

**Figure 1:**
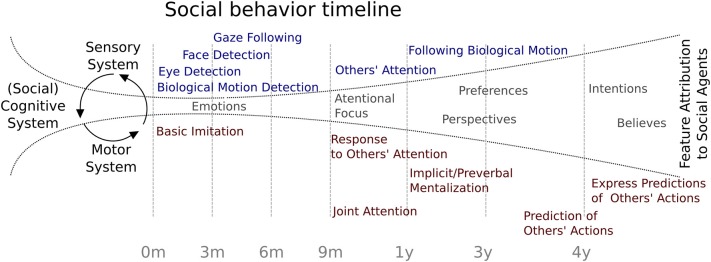
**Social behavior timeline**. Chronology of major social behavior milestones during childhood. Blue indicates evidence related to sensory system maturation and red indicates findings related to motor system maturation. In gray are represented possible feature attributions to social agents.

Considering the evidence reviewed, Figure 1 summarizes the behavioral chronology of the main milestones of social development during the first 4 years of age. At the beginning, the feature attributions to social agents are constrained by the rudimentary specialization of the sensory abilities such as biological motion and eye detection, face recognition, and gaze following. At some point at 3 months of age, the social cognitive system is starting to open up toward the incorporation of the other's attention in a rudimentary interaction. This process may be associated to a neural specialization which the environmental influence has a critical role (see below). At 9 months of age, infants begin to respond to social agent attention and months later, to initiate intentional social interchanges. At 13 months of age, evidence has shown that social attributions begin to be more refined, allowing the child to include other's perspectives, such as preferences, perspectives, intentions, and beliefs, showing the first signs of the ability to make predictions about other people's behavior. Finally, this development becomes more specialized and enables children to predict other's actions and to explicitly express those predictions.

Even though this behavioral evidence has been very informative, there is another valuable area of data that could be useful to elucidate the trajectory of human social development. In order to shed light on the mechanisms that are the basis of human social functioning, we will discuss the findings of the neural processes that underlie social behavior.

## Neural correlates of the development of social skills

Although the development of social behavior is also influenced by a wide variety of hormones, such as oxytocin and vasopressin and steroid hormones like testosterone (for a complete review see McCall and Singer, 2012), the analysis of these factors are beyond the scope of this review. We will focus on cerebral networks that have been associated with social functioning (Wang, 2010; Kennedy and Adolphs, 2012; Billeke and Aboitiz, 2013), especially on evidence emerging from studies conducted with electroencephalography and brain imaging techniques (Wang, 2010).

### Electroencephalographic evidence of the development of social skills

Electroencephalography (EEG) is a technique widely used in human neuroscience because it is a non-invasive technique that allows direct measurements of electrical brain activity from scalp electrodes (DeBoer et al., 2007; Billeci et al., 2013). Especially with infants and children, the EEG is useful tool because it can be informative in absence of an observable behavior, which is often the case with infants (DeBoer et al., 2007). However, the EEG analysis in infants and children is full of difficulties and limitations, due to processes such as myelination, synaptic elimination, increase in skull thickness and fontanel closing, which can influence both amplitude and latency of the event-related potential (ERPs) across different ages (DeBoer et al., 2007). The computational analysis of the EEG signal provides two types of neural activity. The first one is the response evoked by a stimulus or event, namely event-related potential (ERP). ERPs are obtained from the average of several trials with the purpose of eliminating the interference of signals related to the stimulus of interest. Therefore, this methodology analyses brain wave forms that are phase-locked to the stimulus presentation. The second one is the analysis of oscillatory brain activity that is not necessarily phase-locked to the stimulus presentation (Tallon-Baudry and Bertrand, 1999). Indeed, by mean of the study of oscillatory brain activity it is possible to study the brain activity not related to a specific task, namely the study of spontaneous brain activity.

#### Contribution of early ERP components to the study of the development of social skills

The early ERP components usually occur during the first 200 ms after stimuli presentation (McCulloch, 2007), and can serve as markers to follow the functional development of neuronal activity. For example, the early visual component P1 can be used to understand the visual processes involved in the abilities related to social functioning. The P1 component is a positive deflection that arises between 90 and 150 ms after a visual stimulus (Luyster et al., 2014) and is generated in the occipital visual cortex. This component is present in individuals of all ages (Haan et al., 2002), showing modulation by spatial information (Hopf and Mangun, 2000) and low sensitivity to stimulus familiarity (de Haan and Nelson, 1999). In infants and young children, there is an increase in P1 amplitude with age (Luyster et al., 2014). However, sometime between the ages of 4 and 6 years, this pattern is reversed, and the amplitude of the P1 starts to decrease with age (Kuefner et al., 2010), likely reflecting the process of synaptic pruning (Luyster et al., 2014). In accordance with this evidence, the P1 elicited by human faces shows a decrease in its amplitude and latency between the 9 and 17 years of age (Hileman et al., 2011). In fact, typically developing children and young adolescents showed larger P1 amplitudes for inverted faces compared to upright faces (Hileman et al., 2011). Interestingly, in this study the smaller P1 amplitudes were correlated with fewer atypical social behaviors and better social cognitive skills.

Another important ERP component that has been linked with social development is the N170. The N170 component is a negative deflection that peaks between 140 and 170 ms over posterior temporal sites, being elicited by human faces (Courchesne et al., 1981; de Haan and Nelson, 1997, 1999; de Haan, 2002; Haan et al., 2002; Itier, 2004; Dawson et al., 2005; Johnson et al., 2005; de Haan et al., 2007; Csibra et al., 2008; Elsabbagh et al., 2009; Hileman et al., 2011). In adults, this component is generated in the ventral visual pathway, likely from the fusiform face area. This component shows a shorter latency and larger amplitude for faces compared to other stimuli (Haan et al., 2002; Hileman et al., 2011). As in the case of P1, inverted faces elicit larger N170 amplitude than upright faces (Hileman et al., 2011). Several studies have suggested that N170 in infants and children may have a precursor, namely, the N290 component (de Haan et al., 2007; Csibra et al., 2008; Luyster et al., 2014). The infant N290 is a negative deflection that occurs over posterior electrodes of the scalp between 3 and 12 months of age (de Haan et al., 2007; Luyster et al., 2014). This component shows a significant change with age. In a study with infants between 6 and 36 months of age, researchers observed that the N290 component decreases in average amplitude (Luyster et al., 2014). This decrease may reflect the gradual change of this component into N170. The term N290 can be confusing, being called interchangeably the “N290” or a putative “infant 170,” which can be misleading. Nevertheless, Farroni et al. (2002) claim that “putative infant N170” shares some characteristics with the adult N170 component as it is the first negative deflection after the P1 over posterior electrodes. Indeed, after controlling for the impressive change of P1 through time, the infant N170 has in common with the adult component both the latency and the topography since the age of 4 years old (Kuefner et al., 2010). This putative infant N170 component also shows a functional specialization. This orientation effect of N170 (i.e., greater amplitude for inverted faces) becomes evident not before of 6–12 month of age (Haan et al., 2002; Righi et al., 2014). Thus, the developmental change of the N170 component may reveal a cortical specialization during the first year of life (Haan et al., 2002).

Another controversial infant ERP is the Pb component, which it has been associated to social processing, such as JA and emotion perception (Striano et al., 2006; Kopp and Lindenberger, 2011; Jessen and Grossmann, 2014). This component is a positive deflection of early appearance in the frontal and central electrodes which appears between 150 and 250 ms (Striano et al., 2006; Kopp and Lindenberger, 2011; Jessen and Grossmann, 2014). Indeed, the Pb component in infants could correspond to the P2 component in older children and adults (Kopp and Lindenberger, 2011). Pb shows a greater negativity in the condition of JA compared to non-JA (Striano et al., 2006) and is also modulated by face emotion (Jessen and Grossmann, 2014). The Pb component has been interpreted as reflecting stimulus expectancy or contextual processing (Striano et al., 2006; Kopp and Lindenberger, 2011).

#### Contribution of late ERP components to the study of the development of the social skills

The late ERP components are in general described as field potentials that occur 200 ms after the stimulus presentation (Csibra et al., 2008). During childhood, one of the best known late ERP components is the Nc component, which seems to be the first endogenous ERP to emerge in development, being present at birth (Nelson and McCleery, 2008). The Nc component reveals a peak latency decreasing from 800 ms in 1-month-olds (Karrer and Monti, 1995) to 400–600 ms in 1- to 3-year-olds (Goldman et al., 2004; Parker and Nelson, 2005). The peak amplitude of Nc increases with age over the first year of life (Richards, 2003; Webb et al., 2005; Luyster et al., 2014) and then decreases again in the third year of life (Parker and Nelson, 2005; Luyster et al., 2014). The Nc component is considered to reflect attentional orienting to salient stimuli (Courchesne et al., 1981; Pelphrey et al., 2002; Striano et al., 2006) and/or an attentional general activation (arousal), suggesting that children increase their attention to environmental stimuli that are more salient (Striano et al., 2006). Since this component seems to reflect aspects of recognition and familiarity (de Haan et al., 2007), it is elicited in a series of different studies, including face processing. This evidence agrees with the notion that the Nc component is associated to a mandatory attentional processing to a visual stimulus, although not specifically to faces (Luyster et al., 2014). However, the Nc has shown a right-side lateralization, which is consistent with the role of the right hemisphere in the processing of faces (Reynolds and Richards, 2005; Webb et al., 2005; de Haan et al., 2007; Nelson and McCleery, 2008; Luyster et al., 2014). In addition, the Nc component has been widely associated the JA ability (Striano et al., 2006; Kopp and Lindenberger, 2011). In fact, in 9 months old infants, this component shows higher amplitude during JA context than during non-JA contexts in fronto-central channels (Striano et al., 2006; Kopp and Lindenberger, 2011). The neural source of this component has been suggested to be the anterior cingulate cortex (Reynolds and Richards, 2005). Interestingly, in adults, this region together with the right fronto-parietal network participates in the initiation of the joint attention (Caruana et al., 2015).

Another late component observed in infants is the P400. This component is a positive deflection predominantly over right temporo-occipital electrodes, and is more prominent when the stimulus presented a face (de Haan and Nelson, 1999; Haan et al., 2002; Luyster et al., 2014). This component shows a pattern of non-linear age related change, with steadily increasing mean amplitudes between 6 and 24 months and decreasing amplitudes between 24 and 36 months of age (Luyster et al., 2014). Moreover, in the de Haan et al. (2002) study, the infant P400 was observed over occipital and temporal electrodes, elicited by both upright and inverted human and monkey faces. Also, they found that the P400 component showed larger amplitudes for upright than inverted faces, regardless of species, although another study showed the opposite pattern when using familiar faces (Balas et al., 2010). As well as with early ERPs, the evidence of late ERPs components has been suggested that they may be revealing a cortical specialization of the brain during childhood. The evidence reviewed here might be revealed that more voluntary or at least mandatory processes, such as attention or memory, also modulate late ERPs. Thus, neural specialization allows a more efficient stimulus processing thanks to neural resources saving and the capacity of control and redistribution of those resources.

#### Evidence of oscillatory brain activity during the development of social skills

Oscillatory brain activity has been found to participate significantly in social functioning. The oscillatory brain activity is a recurrent brain activity measured in the dimension of time (Klimesch, 2012), that can or cannot be phase-locked to a stimulus presentation. The extracranial EEG signal reflects the neuronal population activity which is commonly decomposed into different frequency ranges namely delta (~2–4 Hz), theta (~4–8 Hz), alpha (~8–12 Hz), beta (~12–30 Hz), and gamma frequencies (~30–100 Hz; Donner and Siegel, 2011).

One of the most studied oscillatory activities in relation to social skills is the mu rhythm. This rhythm occurs in the alpha range between 8 and 12 Hz, but unlike alpha rhythm, which is prominent in the visual cortex, the mu rhythm occurs in the somatic sensorimotor cortex (Oberman et al., 2005; Raymaekers et al., 2009). The mu rhythm amplitude decreases during movement execution and planning, and also during tactile stimulation. Interestingly, mu suppression is also presented during motor imitation and during the observation of other's goal directed movement. Based on this finding it has been proposed that mu suppression can reflect a putative activity of the mirror neuron system (Bastiaansen et al., 2009; Rizzolatti and Sinigaglia, 2010). Mirror neurons were discovered by di Pellegrino et al. (1992) in the monkey premotor cortex. They found that neurons of the rostral part of inferior premotor cortex of the monkey discharges during goal-directed hand movements, such as grasping, holding, and tearing. Mirror neuron system is a special kind of neurons that become active when the monkey performs a particular action and when it observes a similarly performed action by another monkey or human (Gallese et al., 1996). In humans, most of the evidence comes from fMRI or EEG techniques, and results may only be putatively considered as evidence for mirror neuron activity (Buccino et al., 2001; Rizzolatti and Craighero, 2004; Oberman et al., 2005; Iacoboni and Dapretto, 2006; Raymaekers et al., 2009). In spite of this fact, Mukamel et al. (2010) recorded extracellular activity from neurons of 21 patients with pharmacologically intractable epilepsy while they was observing or performing a grasping action or facial gestures. The authors found neurons that responded to both, action-perception and action-execution in two novel brain areas, namely medial frontal cortex and medial temporal cortex (hippocampus, parahippocampal gyrus, and entorhinal cortex). Interestingly, they observed a subset of this kind of cells that increased their firing rate when the subject was in the action-execution condition, but decreased their firing rate when the subject was in the action-perception condition. It is possible to speculate that these neurons can reflect the ability to recognize the differentiation between actions performed by oneself or by someone else (Keysers and Gazzola, 2010).

Although the neural mechanism that connects mu suppression with mirror neurons is still unknown, a plenty of works in adults use this rhythm as a marker of neuron mirror system activity. However, there is yet little evidence about a mu rhythm in children. Lepage and Théoret (2006) obtained data in children between the ages of 4 and 11 years, and found that children showed a mu suppression during the observation of grasping movements (Lepage and Théoret, 2006). Interestingly, using a sample of children between the age of 6 and 17 years, a study found a negative correlation between mu suppression elicited by observing other's movement and the age of the participants (Oberman et al., 2013). This correlation was not found during the execution of the movement. This result reveals a developmental trajectory of mirror neuron systems, possibly related to the specialization of local circuits.

### Imaging evidence of the development of social skills

In addition to EEG, another technique to assess brain development comes from magnetic resonance imaging (MRI) methods and functional MRI (fMRI). The latter imaging technique reflects the changes in hemodynamic brain response related to the neural activity (Auer, 2008) by means of the blood oxygenation level-dependent signal (BOLD) (Ogawa et al., 1990). fMRI has the advantage of a higher spatial resolution, although a lower temporal accuracy related to EEG (de Bie et al., 2012). In recent years, a non-negligible number of evidence has been developed thanks to the use of MRI and fMRI methods to study social skills. Unfortunately, due to technical and ethical issues, fMRI is an intricate method to use with infants and children (Johnson et al., 2005), especially under 4-years old (de Bie et al., 2012).

The infant brain is involved in continuous changes as revealed by several structural imaging studies (Mills et al., 2012). Specifically to the social brain, Mills et al. (2012) describe a developmental trajectory that encompasses several developmental changes of its structures. By the analysis of the structural MRI data of participants between 7 and 30 years old, this study revealed that the gray matter volume and cortical thickness in medial prefrontal cortex (mPFC), temporoparietal junction (TPJ) and posterior temporal sulcus (pSTS) first increases reaching a maximum at about 10 years (on average), and then declines until around age 20 (Mills et al., 2012). These structures have been linked with both theory of mind skills and the prediction of others' behavior (Saxe and Kanwisher, 2003; Saxe et al., 2009; Billeke et al., 2013b, 2014b, 2015). Furthermore, Mills et al. (2012) reported that the volume of gray matter in the anterior temporal cortex increases until adolescence and cortical thickness into young adulthood, which has been also associated with the processing of mentalization (Saxe and Kanwisher, 2003), especially when the use of contextual and prior social information is required (Olson et al., 2012). These changes in brain development can reflect the cortical specialization of the preexisting cerebral structures and networks as a result of the expertise associated with exposure to social environment (Johnson et al., 2005; Johnson, 2011; Davidson and McEwen, 2012).

Regarding mirror neuron system activity, fMRI evidence in adults has revealed that a large number of brain regions are activated during the execution of an action as well as when the same action is seen or heard (Buccino et al., 2001). In children, the mirror neuron system has been linked with mentalizing abilities such as imitating and observing emotional expressions (Iacoboni and Dapretto, 2006). In an fMRI study with children around 10 and 14 years old, Dapretto et al. (2006) observed that the brain regions that were activated during the imitation of emotions were the bilateral striate and extra-striate cortices, primary motor and premotor regions, limbic structures (amygdala, insula and ventral striatum) and the cerebellum. Also, they found a bilateral activity within the pars opercularis of the inferior frontal gyrus (Brodmann's area 44) as well as in the neighboring pars triangularis (Brodmann's area 45), with the strongest peaks in the right hemisphere (Dapretto et al., 2006). This brain region has been identified with mirror properties in adult human (Buccino et al., 2001), showing a possible relationship among imitation and mirror neural networks (Dapretto et al., 2006).

In addition, Saxe et al. (2009) showed that in children between 6 and 11 years, the brain regions involved in perceiving and reasoning about other people were the bilateral TPJ and the precuneus. The mPFC was also active but with a lower threshold than the other brain regions (Saxe et al., 2009). Interestingly, when they examined the possible change related to age, they found that only the right TPJ showed a significant correlation with age, which may reveal a maturational selectivity for social information. Moreover, they observed that the brain regions that were involved in theory of mind processing did not overlap with brain regions devoted to the perception of biological motion. In fact, they found that the perception of biological motion was related to the recruitment of right pSTS. This is a remarkable finding for a full understanding of the social phenomena as a developmental outcome, because it suggests that theory of mind comprehension may rely on a distinct and later developed neural substrate (Saxe et al., 2009).

**Figure 2:**
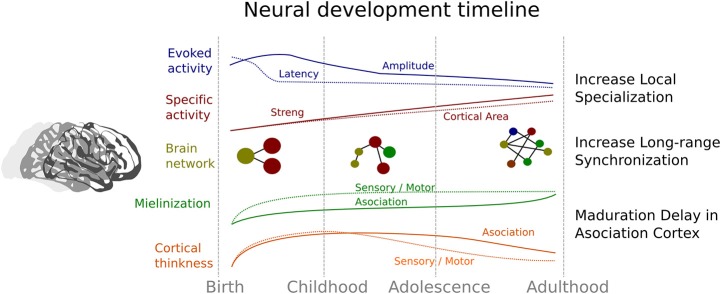
**Summary of the neural evidence related to the developmental trajectory of the social brain**. Blue indicates changes related to event-related potential evidence; Red denotes changes associated with the brain activity related to specific social tasks. Continuous line represents the strength in association between brain activity and social tasks, and dotted line indicates the areas of brain that show significant activity. Yellow represents the change in connectivity and architecture of the brain networks. Green represents changes in myelination and orange changes in cortical thickness in both sensory-motor areas (dotted lines) and association areas (continuous lines).

In summary, cerebral development involves a process of neural specialization that encompasses different levels that are related to each other. This chronology is represented in the Figure 2. All these neural levels show a trajectory characterized a reduction in the cortical area that are recruited in some activity (e.g., reduction of extension of activity in fMRI studies or the amplitude of ERP components), that can reflect an increase in the local efficacy of social processing. Specifically, this developmental trajectory is based on a first increment followed by a decrease of cortical thickness, amplitude, and latency in the ERP, while there is a concomitant increase in myelination and selectivity activity. Indeed, the development of brain networks indicates a decrease or segregation of local connectivity together with an increase in the connectivity between distant brain regions (Fair et al., 2009). Thus, the specialization improvement is also evident in a constant change in the organization of brain networks (Smit et al., 2012; Betzel et al., 2014; Tymofiyeva et al., 2014) that enables the development of an efficient processing lifelong. The evidence reviewed here can shed light on the relationship between brain maturation and the acquisition of social skills. Although the evidence in non-human primates and healthy human beings is remarkable, the study of certain disorders with alterations in social development like autism can be extremely informative and useful (Kennedy and Adolphs, 2012) for a better understanding of the normal developmental trajectory. According to this aim, in the next section we will briefly discuss some the evidence found in ASD, in order to shed light on the development of the social functioning and its neural correlates in these subjects.

## Alterations in the development of social skills: lessons from autism spectrum disorders

ASD are a heterogeneous group of neurodevelopmental disorders that include symptoms in two main areas: (1) deficit in social communication and social interaction and (2) restricted, repetitive patterns of interests, activities, or behavior. These symptoms only become evident after the third year of life (American Psychiatric Association, 2013). This makes it necessary to take into account the need to identify early reliable markers for this disorder, which would also allow earlier detection and more effective interventions (Gliga et al., 2014). The neurobiological evidence consistently indicates that ASD are multifactorial disorders, but unfortunately, their underlying mechanisms are still unknown (Billeci et al., 2013). Some findings imply alterations in the signaling pathways of neurotrophic factors such as BDNF, in dendritic development and synaptic connections, and in vesicular traffic (Chapleau et al., 2009; Penzes et al., 2011; Durand et al., 2012). Other reports indicate differences in the neuroanatomical volumes (Aoki et al., 2012), in mitochondrial function (Rossignol and Frye, 2012), and some others imply the involvement of glia (Ahlsén et al., 1993; Vargas et al., 2005; Aoki et al., 2012).

In addition, the behavioral, electrophysiological, and imaging evidence in children with ASD have reported abnormalities in several social processes. These findings include impairments in the mirror neuron system (Oberman et al., 2005; Dapretto et al., 2006; Raymaekers et al., 2009), in multisensory processing and in its link to complex cognitive functions such as speech (Redcay and Courchesne, 2008; Stevenson et al., 2014), in deficits in face recognition appearing in 10-month old infants (Gunji et al., 2013; Luyster et al., 2014), in eye contact (Klin et al., 2002; Pelphrey et al., 2002; Elsabbagh et al., 2009; Jones and Klin, 2013; Von dem Hagen et al., 2013), in the ability of JA (Charman et al., 2001; Charman, 2003; Morgan et al., 2003; Mundy and Jarrold, 2010; Redcay et al., 2013), in the ability of mentalizing (i.e., the ability to appreciate the difference between the own knowledge and that of the others; Baron-Cohen et al., 1985; Happé, 1995; Charman et al., 2001), and in playing correlates, i.e., the pretended play (Wing and Gould, 1979; Ungerer and Sigman, 1981; Charman et al., 2001).

Furthermore, several reports have described ASD as a disorder of neural synchrony, which has its origins in functional connections within and between brain regions usually mediated by alpha, beta, and theta oscillations (Uhlhaas and Singer, 2006; Righi et al., 2014). The prevailing hypothesis states that ASD is characterized by reduced long-range functional connectivity and increased local functional connectivity (Courchesne and Pierce, 2005; Righi et al., 2014). Several studies in adults and children have pointed to a disorder of brain connectivity as being responsible for abnormal social cognition in ASD, specifically among the components of the social brain (Stroganova et al., 2007; Gotts et al., 2012; Rudie et al., 2012). For example, an abnormal functional coupling between the amygdala and temporal cortex is shown when processing faces (Kleinhans et al., 2008), as well as reduced long-range amygdala connectivity (Rudie et al., 2012). In fact, an interesting study in boys between 3 and 8 years with ASD and typically developing children observed that boys with ASD showed a higher amount of prefrontal delta during stillness and in a sustained visual attention task (Stroganova et al., 2007). They also found an abnormal EEG power asymmetry over the mid-temporal regions. The authors claim that this finding could be interpreted as a reduced neural connectivity in the right temporal cortex which might explain a decreased capacity of the right temporal cortex to generate EEG rhythms. An alteration in long-range functional connectivity has also been demonstrated using fMRI in toddlers with ASD (Dinstein et al., 2011). Although the reduction in long-range connectivity can also be reflected in a decrease of the structural connection between regions (e.g., reduction in corpus callosum volume), a recent study in large sample does not found structural deficit in ASD in corpus callosum (Lefebvre et al., 2015).

Regarding to mirror neurons functioning in ASD, the EEG evidence has been contradictory. Oberman et al. (2005) observed that subjects between 6 and 47 years of age with high-functioning ASD showed a lack of mu suppression while they observed a hand movement but not when they performed the action. Additionally, they found no correlation between age and mu wave suppression in either group. By contrast, Raymaekers et al. (2009) observed no significant differences between high-functioning individuals with ASD and a control group of children aged 8–13 years. Interestingly, the developmental trajectory of the mu rhythms present the same negative correlation with age in ASD and controls, suggesting that local circuit specialization is spared in this condition (Oberman et al., 2013). Nevertheless, the previously referred study of Stroganova et al. (2007) in boys between 3 and 8 years showed differences in mu rhythms between children with ASD and typically developing children during stillness and sustained visual attention task. They observed that the mu rhythm in boys with ASD lacked the leftward asymmetry present in typically developing children. According to the authors, this finding may reveal an abnormal lateralization of sensorimotor function in autism, which might indicate a decreased dominance of the left hemisphere for motor functions in children with ASD. Following these contradictory evidences, in adults with ASD a fMRI study did not find any anomalities in the mirror neurons system activity to observe other people goal-direct behaviors (Dinstein et al., 2010). Hence, it is important to carry out more studies directly addressed to the evolution of these activity during the age.

Other social skill that has been reported altered in ASD is face recognition (Klin et al., 2002; Pelphrey et al., 2002; Gunji et al., 2013; Luyster et al., 2014). In fact, studies have found evidence of Nc right lateralization in young children with ASD. In general, studies have reported two main findings: (1) young children with ASD did not show a differential Nc response to familiar faces vs. unfamiliar faces, or (2) this differential Nc response was delayed relative to typical development. In addition, consistent evidence has been reported that individuals with ASD have abnormal responses to the sensory environment (Baruth et al., 2010). These findings showed that for individuals with autism there may be a sensory overload that can impair their perceptual and cognitive functioning, increase their physiological stress, and adversely affect their social interaction (Baruth et al., 2010). The early visual components related to face processing, such as P1 and N170, also present an alteration in ASD children (Baruth et al., 2010; Hileman et al., 2011). The previously referred study by Hileman et al. (2011) observed that ASD subjects showed a longer N170 latency than individuals with typical development. Furthermore, considering the amplitude of these early components, Luyster et al. (2014) found that in autism high-risk children between 6 and 36 months of age did not evidence a maturational alteration in P1, having similar mean amplitudes than low-risk children. However, they observed a wider difference between groups at later ages. Hileman et al. (2011) also showed that individuals with ASD do not have differential P1 amplitudes for upright and inverted faces. While for typically developing individuals smaller P1 amplitudes were associated with fewer atypical social behaviors and better social cognitive skills, in ASD subjects, there were no relations between the ERP components and atypical social behaviors and social cognition (Hileman et al., 2011).

Evidence like this could be revealing that these ERP differences might be reflecting a low specificity of neuronal and cognitive processes in these children. Indeed, it has been widely reported in EEG, imaging and magnetoencephalograpic literature, that subjects with ASD exhibit reduced functional corticocortical connectivity (Barttfeld et al., 2011; Khan et al., 2013; Nair et al., 2013; Alcauter et al., 2014; Righi et al., 2014). Taking all this evidence into account, it is worth noting that the analysis of social phenomena requires integrative models of the developing social brain that should include both early and late neuronal and cognitive processes. In accordance to this integrative perspective, we will present a blueprint of the main elements that a model of social functioning should take into account, in order to shed light to the development of social processes and its possible alteration in neurological and psychiatric conditions as ASD.

## A specialized brain. a model of the developing social brain

Considering the evidence reviewed here, the establishment of a cognitive model of development of social functioning should consider both, a dynamic perspective that takes into account the temporal dimension and also the constraints imposed by neural and behavioral evidence. Following the proposal of Johnson (2011), the developmental changes in neural processes can represent the specialization of brain functioning to decode social relevant stimuli in order to adapt behavior to a rich social environment. Indeed, recent evidence of brain networks indicates a segregation of local connectivity together with an increase in the connectivity between distant brain regions during development (Fair et al., 2009). The increases in brain network organization (Smit et al., 2012; Betzel et al., 2014; Tymofiyeva et al., 2014) among more specialized brain regions can thus serve as a computational basis for the more complex and flexible behaviors, as demanded by the social environment (Kennedy and Adolphs, 2012; Billeke et al., 2014a). We propose that this specialization can be understood as a general framework that attempts to reduce uncertainty of social environment following a kind of Bayesian inference (Friston, 2010; Koster-Hale and Saxe, 2013). Human beings live in large groups that increase uncertainty of possible consequences of controlling and intervening the behavior of others. In this context, it seems probable that the human brain comes equipped with “social devices,” which allow us to read, interpret and finally, to predict other's behaviors during development. These devices involve primary genetically encoded circuits that presumably required the continue interaction with the environment for their development. We argue that the ability to predict other's actions is possible due to these early onset devices that become more complex and specific through continued interaction with social environment. This does not mean that social cognition is only a prediction achievement, but the prediction of others' behavior is the basis for development of more complex levels of inference (e.g., first and second order mental state attribution). For example, the behavioral evidence reviewed here indicates that children can predict behavior of other people with a false believe (implicit ToM) before being able to verbally express this prediction or being able to give an explanation of other's people behavior (explicit ToM). Indeed, the first trace of the implicit ToM and social perspective takes place when children become able to follow a biological motion (see Figure 1). Thus, these abilities may indicate the first signs of the ability to make predictions about the social world. These abilities evolve so that humans easily learn to infer the intention of others (i.e., building an internal model or internal representation of other) in order to make more accurately predictions of others' behavior. Complex mental state attributions could thus represent a refined mechanism to reduce uncertainty about the social environment.

**Figure 3:**
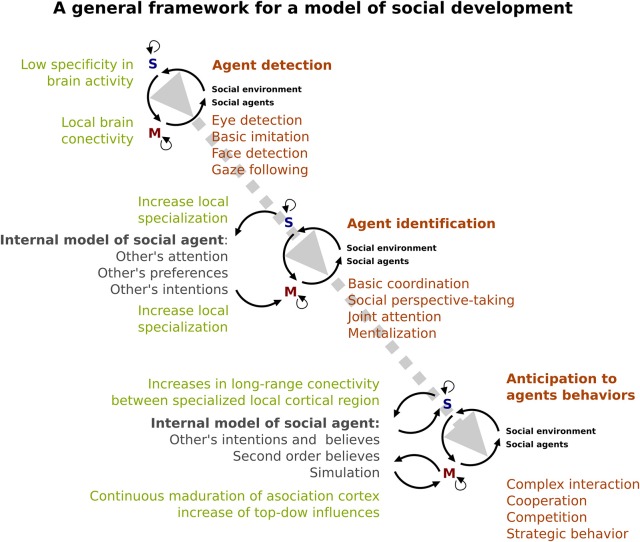
**A specialized brain**. A model of the developing social brain. Dotted gray line represents the interaction between neural (light green) and behavioral (orange) development. Note that the gray arrow shows an increase in the complexity of that interaction across ages. Dark green shows the emergence and complexity of the internal cognitive model of the social agent. Black lines represent the relationship between sensory (blue S) and motor systems (red M).

The development of an internal model of others could come precisely from interaction between the different early onset “devices” recruited primary to identify social agent and the social environment. As we have described above (see Figures 2, 3), these social devices consist in both sensory and motor mechanisms. The sensory device (“S” in Figure 3) allows us to have the early capacity to distinguish the social agent (e.g., identified eye-like stimulus and other's movement). On the other hand, motor devices (“M” in Figure 3) prepare the infant to interact with a social environment in order to imitate basic motor behaviors of social agents at an initial stage and subsequently, to respond and to be able to coordinate with him/her (e.g., responding joint attention). These devices progressively specialize by interacting with the surrounding environment. Evidence of this specialization is the increased complexity of social behavior from a discrimination of social agents to the inference of their intentions. For instance, the ability of infants to discriminate biological motion from non-biological motion could be the beginning (in time and level of specificity) of the posterior capacity of discriminating between an animal and a human, and later the ability to recognize a familiar/unfamiliar human face. In accordance with this, the EEG evidence suggested that human face sensitivity may experience a cortical specialization during childhood (e.g., Haan et al., 2002; Kuefner et al., 2010) as both amplitude and latency of ERPs changes during infancy and childhood. Interestingly, the developmental changes of brain structures (i.e., changes in gray matter volume, and cortical thickness) can reflect this process of neural specialization (Mills et al., 2012).

We hypothesize that social development depends on a process of neural specialization in these sensory and motor devices. These processes might reveal the development of early onset sensory-motor devices that might work as tools that increase efficiency in the interpretation, attribution, and ultimately, prediction of the behavior of the social agents in order to engage in complex social interactions (e.g., cooperation, competition, bargaining, etc.). The development of the internal model of social agents imply the consolidation of previous experiences and the organization of those experiences in a complex and flexible way, as well as the development of other cognitive abilities such as working memory and language. According to the evidence reviewed here, the capacity to create an internal model of social agents could be affected in ASD and may be the basis of the impaired social interaction that is the core of this disorder. This alteration can be understood as a specialization disturbance (Courchesne and Pierce, 2005), as suggested by the evidence indicating a reduced long-range functional brain connectivity and an increased local functional brain connectivity in ASD (Courchesne and Pierce, 2005; Happé and Frith, 2006). Moreover, the EEG evidence that shows alterations in early visual ERPs in ASD (Baruth et al., 2010; Hileman et al., 2011) may indicate a detour in the trajectory of the local circuit specialization. Recent findings revealed that ASD showed impairments in both, automatic neuronal prediction (Dunn et al., 2008) and ability to manage environmental uncertainty (Favre et al., 2015). Thus, following the general framework proposed here, the social alteration in ASD could be understood as a consequence of both, an impairment to accurately make prediction of social agents behaviors, and the ability to adapt their behavior to uncertainty social environments. Furthermore, the pervasive feature of ASD could be a sign of a neural alteration that begins at very early stages of development. However, further studies are necessary to unravel the causal relationship between neural alterations and social impairments in developmental disorders such as ASD.

## Conclusion

The elaboration of cognitive frameworks of social development should take into account the temporal perspective of biological and behavioral changes. In this way, these frameworks can help to elaborate appropriate educational and clinical approaches. Thus, further research in pervasive neurodevelopment diseases such as ASD, should consider integrative approaches, which include the understanding that social development is a complex and large unit between the subject and his/her environment. Hence, we here described the behavioral and neuronal trajectory of the developmental changes related to maturation of social skills during the first years of life. Constrained by these findings, we propose a basic scheme of a possible cognitive model. This model involves the development of an internal template of social agents. Such a process entails the elaboration of efficient prediction of others' behavior, in order to engage in complex social interactions. These processes require the specialization of neural networks to process large amounts of sensory and motor information existing in social environment, in order to be able to perceive, to process, to remember and to discriminate information, with the purpose of predicting and finally understanding others.

### Conflict of interest statement

The authors declare that the research was conducted in the absence of any commercial or financial relationships that could be construed as a potential conflict of interest.
